# Cathode Properties of Na_3_FePO_4_CO_3_ Prepared by the Mechanical Ball Milling Method for Na-ion Batteries

**DOI:** 10.1038/s41598-020-60183-3

**Published:** 2020-02-24

**Authors:** Baowei Xie, Ryo Sakamoto, Ayuko Kitajou, Kosuke Nakamoto, Liwei Zhao, Shigeto Okada, Yuki Fujita, Nobuto Oka, Tetsuaki Nishida, Wataru Kobayashi, Masaki Okada, Toshiya Takahara

**Affiliations:** 1 Interdisciplinary Graduate School of Engineering Science, Kyushu University, 6-1, Kasuga Koen, Kasuga, 816-8580 Japan; 2 Organization for Research Initiatives, Yamaguchi University, 2-16-1 Tokiwadai, Ube, 755-8611 Japan; 3 Institute of Materials Chemistry and Engineering, Kyushu University, 6-1, Kasuga Koen, Kasuga, 816-8580 Japan; 4 Department of Bioenvironmental Chemistry, Kindai University, Iizuka, Fukuoka Japan; 5 Tosoh Corporation, 3-8-2, Shiba, Minato-Ku, 105-0014 Tokyo, Japan

**Keywords:** Batteries, Energy

## Abstract

The carbonophosphate Na_3_FePO_4_CO_3_ was synthesized by the mechanical ball milling method for the first time. The composition of the obtained sample with a higher amount of Fe^2+^ was Na_2.66_Fe^2+^_0.66_Fe^3+^_0.34_PO_4_CO_3_ as confirmed by Mössbauer analysis, owing to the good airtight properties of this method. The obtained samples in an organic electrolyte delivered an initial discharge capacity of 124 mAh/g at room temperature, and a larger discharge capacity of 159 mAh/g (1.66 Na^+^/mole) at 60 °C. With 17 m NaClO_4_ aqueous electrolyte, a discharge capacity of 161 mAh/g (1.69 Na^+^/mole) was delivered because of the high ionic conductivity of the concentrated aqueous electrolyte. During the charge-discharge process, the formation of Fe^4+^ after charging up to 4.5 V and the return of Fe^2+^ after discharging down to 1.5 V were detected by *ex-situ* X-ray absorption near edge structure (XANES) analysis.

## Introduction

Sodium as a charge carrier, is one of the most abundant elements on earth. Infinite sodium amounts could be exploited from the ocean, which makes Na-ion batteries (NIBs) a good cost-performance alternative for large-scale grid storage applications with low environmental impact. Compared with the layered oxide cathodes, which readily release oxygen when charged, polyanionic cathodes are more promising as an energy storage system in terms of structural stability, operating voltage, and safety.

For the past decades, various iron-based polyanionic compounds such as NAtrium Super Ionic CONductor (NASICON)-type Fe_2_(SO_4_)_3_^1^, Na_2_FePO_4_F^2^, NaFeSO_4_F^3^, olivine-type NaFePO_4_^4^, Na_2_FeP_2_O_7_^5^, NaFe(PO_3_)_3_^6^, and alluaudite-type Na_2_Fe_2_(SO_4_)_3_^7^ have been extensively studied for their potential as cathode materials for NIBs. However, their capacities are limited to one-electron reaction with Fe^2+^/Fe^3+^ as the redox couple, and thus their capacities are not comparable with the layered oxide cathodes (e.g. NaFeO_2_^8^). Recently, a new compound with a monoclinic structure, Na_3_FePO_4_CO_3_, has been researched as a favorable cathode, due to its high theoretical capacity of 192 mAh/g utilizing Fe^2+^/Fe^3+^ and Fe^3+^/Fe^4+^ during cycling^9^. However, the energy barrier between the Na diffusion paths is 0.6 eV, which will lead to a low ionic conductivity. Although Na_3_FePO_4_CO_3_ was synthesized experimentally by a hydrothermal method^10^, the operations would take place in air (such as: washing with water in air), making it difficult to prevent the oxidation of Fe^2+^. This problem must be overcome before the electrochemical performance of Na_3_FePO_4_CO_3_ can be improved.

Due to its high efficiency and convenience, high energy mechanical ball milling has been used to synthesize nanostructured cathode and anode materials for lithium-ion batteries (LIBs) and NIBs^11–13^. By increasing the surface area of the nanoparticles, the electrochemical performance of these samples could be greatly enhanced. In addition, the mechanical ball milling method allows working in complete insert atmosphere, which prevents oxidation of the final products. Therefore, we synthesized nano-size Na_3_FePO_4_CO_3_ by the mechanical ball milling method. Its electrochemical properties as a cathode for NIBs were evaluated in an organic electrolyte at ambient temperature and 60 °C, respectively.

The rate capacity of NIBs in organic electrolytes is limited by the slower sodium transport because of the larger ion size of sodium ion. In addition, due to the use of flammable organic electrolytes, safety problems are a concern for application to large-scale grid storage. On the other hand, aqueous electrolytes are non-flammable and show better ionic conductivity, which makes them as a good alternative for large-scale grid storage. After the first concept of aqueous electrolytes for LIBs^14,15^, it has been explored for application to NIBs^16–20^. However, traditional aqueous electrolytes have suffered from narrow electrochemical potential windows, especially in diluted solutions. Increasing the salt concentration usually results in dramatic improvement of the operation voltage in aqueous electrolytes^19,20^. As a practical anode for aqueous NIBs, NaTi_2_(PO_4_)_3_ with a NASICON framework has been reported to be an efficient anode in 2 M Na_2_SO_4_ aqueous electrolytes^17^. The Prussian-blue type of Na_2_MnFe(CN)_6_^21^ and the polyanionic cathodes of NaVPO_4_F^20^, Na_2_FeP_2_O_7_^22^, Na_3_V_2_O_2x_(PO_4_)_2_F_3-2x_^23^, and Na_3_MnPO_4_CO_3_^24^ are studied as cathodes for aqueous NIBs. K. Nakamoto and co-workers reported that highly concentrated aqueous electrolyte of 17 m NaClO_4_ shows stable the electrochemical potential window up to 2.78 V, which exceeds the theoretical operating voltage of 1.23 V for the aqueous batteries^25^. In this study, we also explore the cathode properties of a full cell Na_3_FePO_4_CO_3_ // NaTi_2_(PO_4_)_3_ in the aqueous electrolyte of 17 m NaClO_4_.

## Results and Discussion

**Figure 1:**
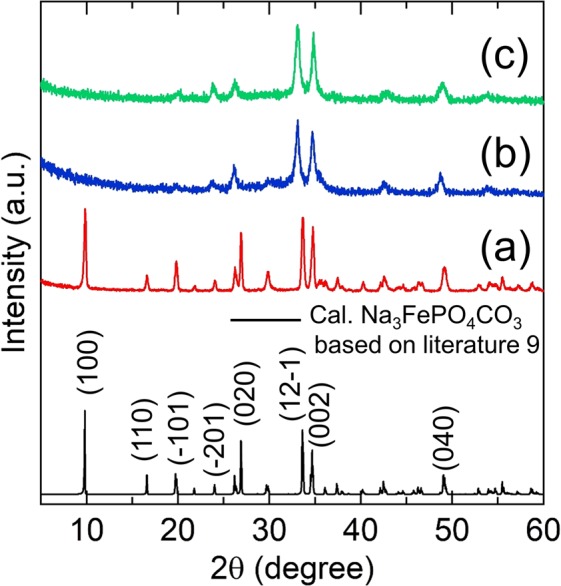
XRD patterns of Na_3_FePO_4_CO_3_ synthesized by the hydrothermal method and denoted as HD_NFPC (**a**); Na_3_FePO_4_CO_3_ synthesized by the mechanical ball milling method and denoted as MM_NFPC (**b**); HD_NFPC ball milled at 600 rpm 12 h (**c**).

Diffraction peaks of maricite-NaFePO_4_ synthesized by the solid state method^26^ were indexed to the Pnma space group NaFePO_4_ (Inorganic Crystal Structure Database (ICSD) card no. 25–0770) (Fig. S1). X-ray diffractometer (XRD) patterns of Na_3_FePO_4_CO_3_ synthesized by the hydrothermal method and denoted as HD_NFPC (Fig. 1(a)), Na_3_FePO_4_CO_3_ synthesized by the mechanical ball milling method and denoted as MM_NFPC (Fig. 1(b)), and HD_NFPC ball milled at 600 rpm for 12 h (Fig. 1(c)) are summarized in Fig. 1. The monoclinic Na_3_FePO_4_CO_3_ with the space group P2_1_/m (ICSD card no. 07–7053) was defined from the XRD pattern of HD_NFPC (Fig. 1(a)). However, the peak near 10° resulting from (1 0 0) reflection disappeared and the crystal plane of (1 2 −1) shifted to the low angle side in the sample of MM_NFPC (Fig. 1(b)). With respect to the disappearance of (1 0 0), a similar phenomenon can be detected for Na_3_MnPO_4_CO_3_/Na_3_FePO_4_CO_3_ after ball milling, because of the tendency for the Mn or Fe cation to occupy the Na cation^27,28^. With respect to the shift of (1 2 −1), this could be associated with changes of the lattice parameters after ball milling^28^. Table 1 presents the lattice constants for Na_3_FePO_4_CO_3_ (ICSD card) and the obtained products of MM_NFPC. The XRD pattern of HD_NFPC ball milled at 600 rpm for 12 h (Fig. 1(c)) also showed the same changes as in Fig. 1(b), suggesting that the structural changes occurred from the high energy ball milling process. In addition, the XRD curve of MM_NFPC was consistent with simulated diffraction peaks from the refined lattice parameters in Table 1 (Fig. S2), which also indicated formation of Na_3_FePO_4_CO_3_ after mechanical ball milling.

**Table 1:** The lattice parameters for the ICSD card and the MM_NFPC sample.

	a [Å]	b [Å]	c [Å]	α[^°^]	β[^°^]	γ[^°^]	Space group
ICSD Card NFPC	8.95	5.15	6.63	90	90	90.45	P2_1_/m
MM_NFPC	8.87	5.16	6.81	90	90	90.54	P2_1_/m

**Figure 2:**
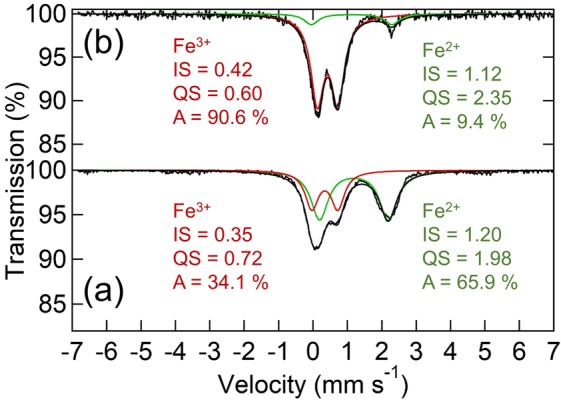
^57^Fe Mössbauer spectroscopy of the MM_NFPC/C kept in an Ar-filled glovebox (**a**); exposed in air (**b**).

The results of ^57^Fe Mössbauer spectroscopy of the MM_NFPC/C kept in an Ar-filled glovebox are presented in Fig. 2(a). The spectra consisted of two symmetric doublets, showing that the matrixes after mechanical ball milling had 65.9 atom% of Fe^2+^ and 34.1 atom% of Fe^3+^. The composition of MM_NFPC/C was rewritten as Na_2.66_Fe^2+^_0.66_Fe^3+^_0.34_PO_4_CO_3_. Due to the better airtight property, oxidation of Fe^2+^ could be dramatically decreased to 34 atom% per formula unit, which was better than the result of Na_2.24_Fe^2+^_0.24_Fe^3+^_0.76_PO_4_CO_3_ by the hydrothermal method^10^. For the MM_NFPC samples exposed in air (Fig. 2(b)), the fraction of Fe^2+^ decreased to 9.4 atom% per formula unit due to its higher sensitivity to O_2_ in air, which was also detected by N. Kosova and co-workers^28^.

**Figure 3:**
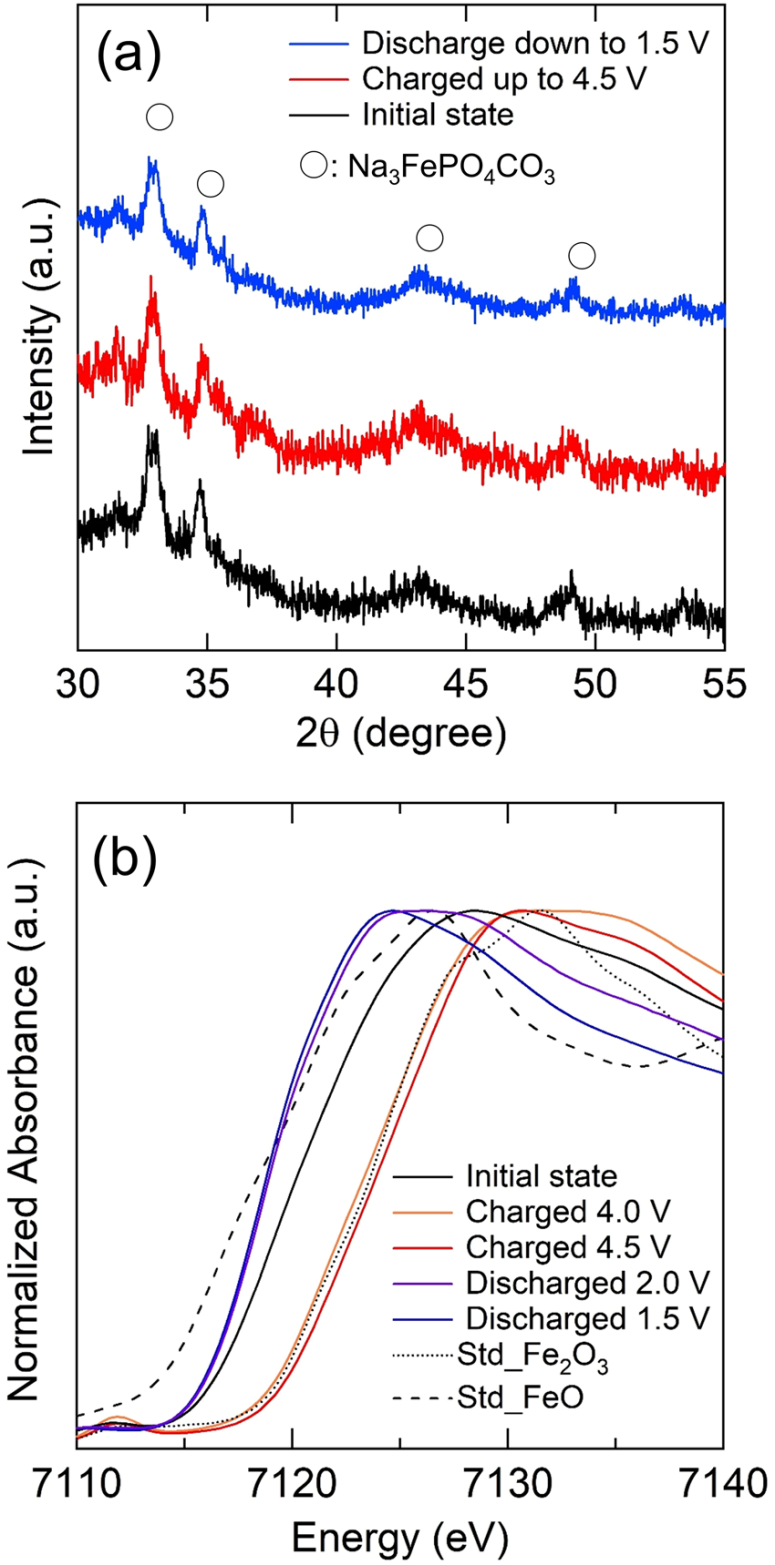
*Ex-situ* XRD patterns of MM_NFPC/C at different states during the first cycle between 1.5 and 4.5 V at 0.2 mA/cm^2^ at room temperature (**a**); *ex-situ* XANES spectra of MM_NFPC/C at different states during the first cycle between 1.5 and 4.5 V at 0.2 mA/cm^2^ at room temperature (**b**).

The results of *ex-situ* XRD measurements of MM_NFPC/C at the initial state, charging up to 4.5 V and discharging down to 1.5 V, are shown in Fig. 3(a). The main peak for the samples in the different states was well maintained at 33.1° and 34.8°, indicating that the structure had good stability during the charge-discharge process. *Ex-situ* XANES measurements were carried out at different cutoff voltages to gain a deeper understanding of the mechanism of the two-electron process of Fe^2+^/Fe^4+^ in the intercalation and deintercalation reaction. The results of the *ex-situ* XANES for different states of cathodes are shown in Fig. 3(b). The spectra of the initial state MM_NFPC/C exceeded the standard sample of FeO, suggesting that Fe^2+^ was partially oxidized during the synthesis process, which was consistent with the results of the Mössbauer analysis. The near-edge shifted to a higher energy side near the Fe_2_O_3_ spectra after charging up to 4.0 V, and a more obvious shift to the right side beyond the spectra of the Fe_2_O_3_ was observed after further charging up to 4.5 V, suggesting the oxidation of Fe^2+^ to Fe^4+^. The near-edge overlapped with the spectra of FeO, after discharging down to 2.0 V and fully discharging down to 1.5 V, suggesting the reduction of Fe^2+^ during the intercalation process.

**Figure 4:**
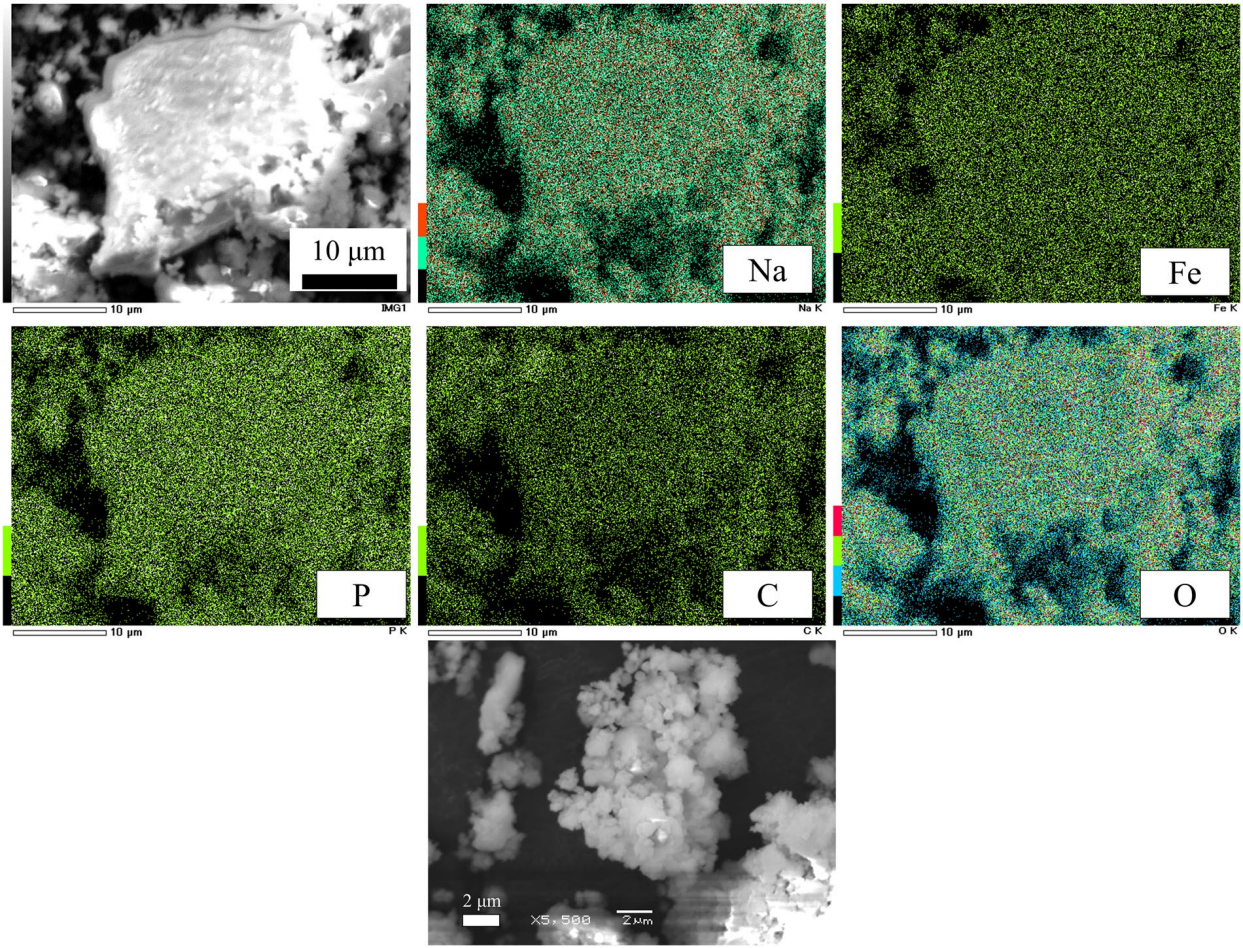
EDX mapping and SEM images of the MM_NFPC.

The distributions and the morphological characteristics of each element, as determined by scanning electron microscopy (SEM) and energy dispersive X-ray spectrometry (EDX), are shown in Fig. 4. As seen in the EDX mapping, the elements of Na, Fe, C, O and P were distributed uniformly in MM_NFPC particles. In SEM images, ~500 nm particles had an irregular shape due to powerful milling. Because the formation of nano-size particles can increase the contact area between the cathode and electrolyte, excellent cathode properties of the obtained sample can be expected.

The thermal gravimetric (TG) curves of MM_NFPC and HD_NFPC are colored red and black, respectively, in Fig. S3. The weight loss in the range of 50 °C to 250 °C was associated with the physically absorbed water, while at higher temperatures it was related to the release of CO_2_ decomposed from Na_3_FePO_4_CO_3_. For the black line, the weight loss was 13.0 wt% in the temperature region of 480 °C to 600 °C, and the decomposition temperature was near 530 °C. For the red line, the weight loss was about 13.3 wt%, which was close to the theoretical mass loss value of 14.8 wt% in the temperature region from 300 °C to 550 °C. And the decomposition temperature was near 380 °C, which was lower than that for the sample synthesized by the hydrothermal method. The lower temperature was attributed to the presence of structural defects during the ball milling process.

**Figure 5:**
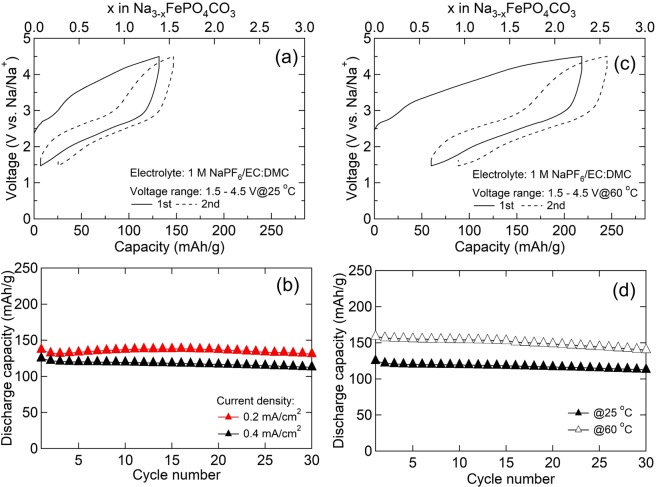
The initial charge-discharge curves obtained between 1.5 and 4.5 V at the current density of 0.4 mA/cm^2^ at 25 °C (**a**); the cyclability is on the bottom side (**b**); the initial charge-discharge curves obtained between 1.5 and 4.5 V at the current density of 0.4 mA/cm^2^ at 60 °C (**c**); the cyclability is on the bottom side (**d**).

The initial charge-discharge curves and the cyclabilities of MM_NFPC/C obtained from 1.5–4.5 V at 0.4 mA/cm^2^ at room temperature are shown in Fig. 5(a). At the first charging, a capacity of 131 mAh/g (1.38 Na^+^/mole) exceeding the one-electron reaction capacity (96 mAh/g) was obtained, suggesting that Fe^2+^/Fe^3+^ and Fe^3+^/Fe^4+^ are active during the deintercalation reaction. Meanwhile, two plateaus at 2.7 and 4.0 V associated with Fe^2+^/Fe^3+^ and Fe^3+^/Fe^4+^ were observed. Following the discharge process, the first discharge capacity for MM_NFPC/C was 124 mAh/g (1.30 Na^+^/mole). After 30 cycles, the discharge capacity nearly stabilized at 113 mAh/g and capacity retention was 91%. When the current density was decreased at 0.2 mA/cm^2^, the specific discharge capacity was delivered at 136 mAh/g (1.43 Na^+^/mole) at the first cycle and stabilized at 131 mAh/g after 30 cycles (Fig. 5(b)). Based on the formulation of Na_2.66_FePO_4_CO_3_, the first charge capacity could be about 159 mAh/g, as calculated from the extraction of 1.66 moles of sodium ion per formula unit. To deliver a larger charge capacity, we increased the cutoff voltage from 4.5 to 4.8 V. A larger charge capacity of 165 mAh/g (1.73 Na^+^/mole) and discharge capacity of 141 mAh/g (1.48 Na^+^/mole) were obtained (Fig. S4). However, the irreversible reaction of electrolyte decomposition occurring at a higher voltage would also be attributed to this charge capacity, which would also affect the cyclability of MM_NFPC/C. As a result, due to polarization, the discharge capacity nearly degenerated to 96 mAh/g and the capacity retention was only 68% after 30 cycles. We also investigated the cathode properties at 60 °C between 1.5 and 4.5 V at 0.4 mA/cm^2^. The initial charge-discharge curves are shown in Fig. 5(c). Because of the influence of the kinetics, the discharge capacity was improved from 124 mAh/g to 159 mAh/g (1.67 Na^+^/mole). To the best of our knowledge, this is the first report of such a large capacity for an NFPC cathode. After 30 cycles, the capacity was 140 mAh/g and the capacity retention remained near 88.0%.

**Figure 6:**
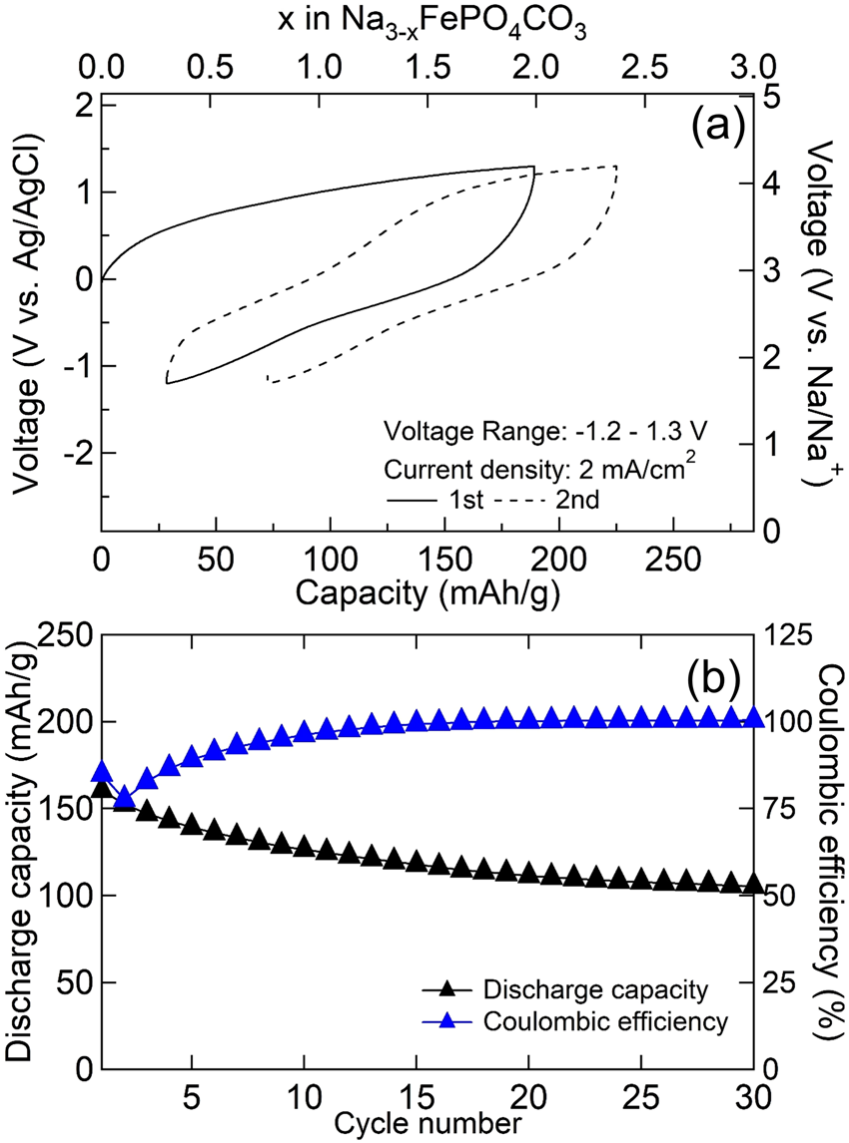
Charge-discharge curves of MM_NFPC/C vs. NTP/C with 17 m NaClO_4_ electrolyte at room temperature (**a**); the cyclability is on the bottom side (**b**).

The XRD pattern from NaTi_2_(PO_4_)_3_ (denoted as NTP) was identified as ICSD no.08–0426 NaTi_2_(PO_4_)_3_ (Fig. S5). In order to evaluate the potential application to large-scale energy storage, the electrochemical performance of a full cell MM_NFPC/C // NTP/C using an aqueous electrolyte of 17 m NaClO_4_ was evaluated at −1.2–1.3 V vs. Ag/AgCl at a current density of 2 mA/cm^2^ (a rate of 0.5 C), at room temperature. The initial charge-discharge curves of the full cell are shown in Fig. 6. A larger discharge capacity of 161 mAh/g was obtained, owing to the high ionic conductivity value of 17 m NaClO_4_ (108 mS/cm^29^), which was better than that of NaPF_6_/EC:DMC (1:1 v/v) (6.5 mS/cm^30^). After 30 cycles, the discharge capacity was 105 mAh/g and the capacity retention was kept at 65.6%. To the best of our knowledge, no report has been published about Na_3_FePO_4_CO_3_ using an aqueous electrolyte and delivering such a large discharge capacity. The specific discharge capacity of MM_NFPC/C // NTP/C with 17 m NaClO_4_ is much better than those of the reported cathodes in aqueous sodium ion batteries, including the full cells of Na_4_Mn_9_O_18_ // NTP^19^, Na_2_FeP_2_O_7_ // NTP^22^, Na_3_V_2_O_2x_(PO_4_)_2_F_3-2x_ // NTP^23^, Na_2_MnFe(CN)_6_ // NTP^25^, and Na_2_CoFe(CN)_6_ // Na_2_MnMn(CN)_6_^31^.

## Conclusion

In summary, Na_3_FePO_4_CO_3_ was synthesized by the mechanical ball milling method. MM_NFPC/C delivered a specific discharge capacity of 124 mAh/g and 159 mAh/g at 25 °C and 60 °C at the first cycle between 1.5 and 4.5 V at 0.4 mA/cm^2^, and it maintained good capacities of 91% and 88% after 30 cycles, respectively. Meanwhile, a full cell MM_NFPC/C // NTP/C using an aqueous electrolyte of 17 m NaClO_4_ was tested for the first time. It delivered a discharge capacity of 161 mAh/g at the first cycle and remained at 105 mAh/g after 30 cycles. From the results of *ex-situ* XANES analysis, the formation of Fe^4+^ after charging up to 4.5 V and the return of Fe^2+^ after discharging down to 1.5 V were confirmed. Reversible structure evolution and an Fe K-edge position shift during the charge-discharge process contributed to the excellent capacity retention of MM_NFPC/C.

## Methods

### Synthesis of NaFePO_4_ (maricite)

The synthesis of NaFePO_4_ was conducted using a solid-state method. First, we mixed Na_2_CO_3_ (Kishida Chemical Co., Ltd.), FeC_2_O_4_·H_2_O (Wako Pure Chemical Industries) and (NH_4_)_2_HPO_4_ (Wako Pure Chemical Industries) in a molar ratio of 1:2:2, and then transferred the mixture into a container with 3 mm diameter ZrO_2_ balls. The mixture was ball milled by the wet planetary ball milling method at 600 rpm for 1 h. Next, the mixture was dried and ground, pressed into a pellet, and annealed at 600 °C in Ar for 8 h.

### Synthesis of Na_3_FePO_4_CO_3_ (mechanical ball milling method)

First, we mixed NaFePO_4_^26^ (maricite) synthesized by the solid-state method and Na_2_CO_3_ (Kishida Chemical Co., Ltd.) in a molar ratio of 1:1, then transferred the mixture to a container with zirconia balls (diameter: 3 mm). NaFePO_4_ and Na_2_CO_3_ were ball-milled using a planetary mill (Pulverisette 7, Fritsch) at 600 rpm for 12 h, and denoted as MM_NFPC. Then we mixed MM_NFPC with 25 wt% of acetylene black (AB, Denka Co., Ltd) and ball-milled at 500 rpm for 6 h. The mixture was ball-milled again with 25 wt% AB at 400 rpm for 4 h to reach a complete weight ratio of Na_3_FePO_4_CO_3_: AB of 60:30, and denoted as MM_NFPC/C. The ball milling process was carried out in an Ar-filled container

### Synthesis of Na_3_FePO_4_CO_3_ (hydrothermal method)

First, 0.002 mol FeSO_4_·7H_2_O (Kishida Chemical Co., Ltd.) was dissolved into 5 ml of deionized water to form a clear solution A. Secondly, 0.002 mol (NH_4_)_2_HPO_4_ (Wako Pure Chemical Industries) and 2 g Na_2_CO_3_ (Kishida Chemical Co., Ltd.) were dissolved in 10 ml deionized water to form a clear solution B. The solution A and solution B were mixed quickly under magnetic stirring and transferred into an autoclave. Then the autoclave was heated at 120 °C for 20 h. The final product was washed by deionized water and dried at 40 °C in a vacuum oven.

### Synthesis of NaTi_2_(PO_4_)_3_

A stoichiometric mixture of TiO_2_ (Sigma Aldrich), NH_4_H_2_PO_4_ (Wako Pure Chemical Industries) and Na_2_CO_3_ (Kishida Chemical Co., Ltd.) was ball milled at 400 rpm for 1 h, using 3 mm diameter ZrO_2_ balls. This mixture was dried and ground, then made into pellets and annealed at 800 °C for 12 h in air.

### Electrochemical test

The electrochemical test was performed in a non-aqueous electrolyte system as follows. Cathode electrodes were fabricated by mixing 90 wt% MM_NFPC/C with 10 wt% polytetrafluoroethylene (PTFE, Daikin Industries Ltd.) and then pressed into disks (loading amount: 30 mg/cm^2^). After drying the cathode pellet at 120 °C for 12 h under vacuum, the cathode properties were investigated in a 2032 coin-type cell with an organic electrolyte of 1 M NaPF_6_/ethylene carbonate (EC): dimethyl carbonate (DMC) (1:1 in volume, Tomiyama Pure Chemical Industries). All coin cells were fabricated using a glass fiber separator (GA-55, Advantec), and Na metal (Sigma Aldrich) as the anode electrode in an Ar-filled glove box.

For the electrochemical test in 17 m NaClO_4_ aq., cathode electrodes were assembled by mixing 90 wt% MM_NFPC/C with 10 wt% PTFE and then pressed into disks with the same loading of 30 mg/cm^2^. NASICON-type NaTi_2_(PO_4_)_3_ (denoted as NTP) as anode was synthesized by the solid-state method^25^, and then mixed NTP with AB and PTFE in a weight ratio of 70:25:5. The cathode and anode pellets were assembled using Ti mesh as current collector (Thank Metal Co., Ltd.), 17 m NaClO_4_ solution as the aqueous electrolyte (molality (m) = mole of solute/weight of solvent), and a silver-silver chloride electrode with saturated KCl (RE-6, BAS Inc.) as a reference electrode.

### Material characterization

The particle size, morphology, and element distribution were accomplished by SEM and EDX mapping (JSM-6340F, JEOL Ltd.). The thermal properties measurements of MM_NFPC were accomplished under an Ar-flow atmosphere with a Thermo Plus TG-DSC 8230 L system (Rigaku Corp.) at a heating speed of 5 °C/min with Al_2_O_3_ as a reference material. Cathode pellets for the *ex-situ* XRD and *ex-situ* XANES analysis were disassembled from the coin cells after charge/discharge test with 1 M NaPF_6_/EC:DMC (1:1 in volume). A ^57^Fe Mössbauer spectrometer (Laboratory Equipment Corp.) was used with α-Fe foil as a reference for isomer shift measurement. For Mössbauer measurement, the initial cathode pellet was laminated with an aluminum bag in insert atmosphere. The cathode pellets used for the *ex-situ* XRD measurements (50 kV and 300 mA, Cu-Kα radiation, Rigaku Corp.) were disassembled, washed, immersed in DMC liquid, then dried overnight to remove the organic solvents. They were transferred to sample holders for XRD measurements (Rigaku) in insert atmosphere. The *ex-situ* XANES analysis of the iron K-edge were carried out using synchrotron radiation at the BL11 beamline of Saga Light Source.

## Supplementary information

Supporting information.
