# Supplementary material for: Cathode Properties of Na3FePO4CO3 Prepared by the Mechanical Ball Milling Method for Na-ion Batteries
Source: Sci Rep. 2020 Feb 24;10:3278. doi: 10.1038/s41598-020-60183-3 (PMC7039986; doi:10.1038/s41598-020-60183-3)
Supplement: Supplementary file 1 — Supporting information. [file 41598_2020_60183_MOESM1_ESM.docx]

**Cathode Properties of Na_3_FePO_4_CO_3_ Prepared by the Mechanical Ball Milling Method for Na-ion Batteries**

Baowei Xie^a^, Ryo Sakamoto^a^, Ayuko Kitajou^b^, Kosuke Nakamoto^c^, Liwei Zhao^c^, Shigeto Okada^c*^, Yuki Fujita^d^, Nobuto Oka^d^, Tetsuaki Nishida^d^, Wataru Kobayashi^e^, Masaki Okada^e^, Toshiya Takahara ^e^

*^a^ Interdisciplinary Graduate School of Engineering Science, Kyushu University, 6-1, Kasuga Koen, Kasuga, 816-8580, Japan*

*^b^ Organization for Research Initiatives, Yamaguchi University, 2-16-1 Tokiwadai, Ube 755-8611, Japan*

*^c^ Institute of Materials Chemistry and Engineering, Kyushu University, 6-1, Kasuga Koen, Kasuga, 816-8580, Japan*

*^d^ Department of Bioenvironmental Chemistry, Kindai University, Iizuka, Fukuoka. Japan*

*^e^ Tosoh Corporation, 3-8-2, Shiba, Minato-Ku,* *105-0014, Tokyo, Japan*

**Supporting information**


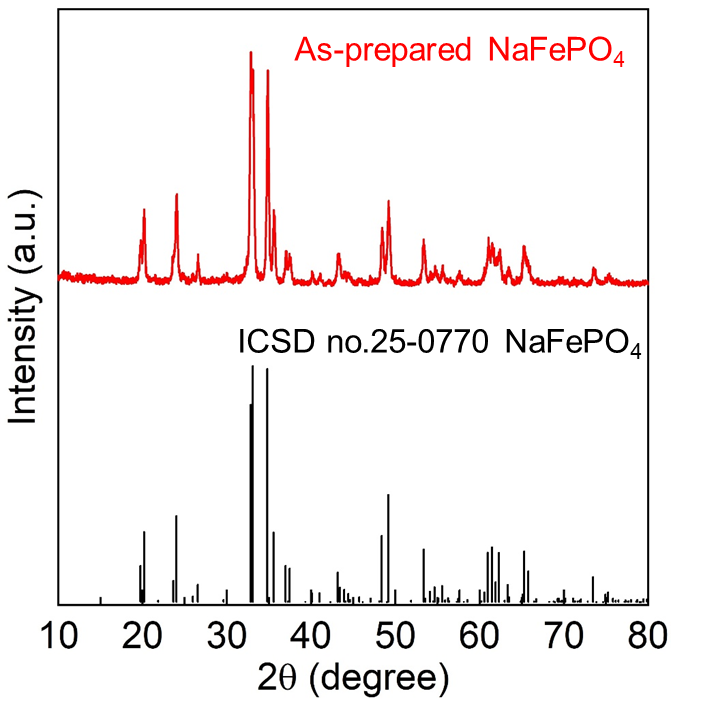


Figure S1. XRD patterns of NaFePO_4_ prepared by solid state reaction.


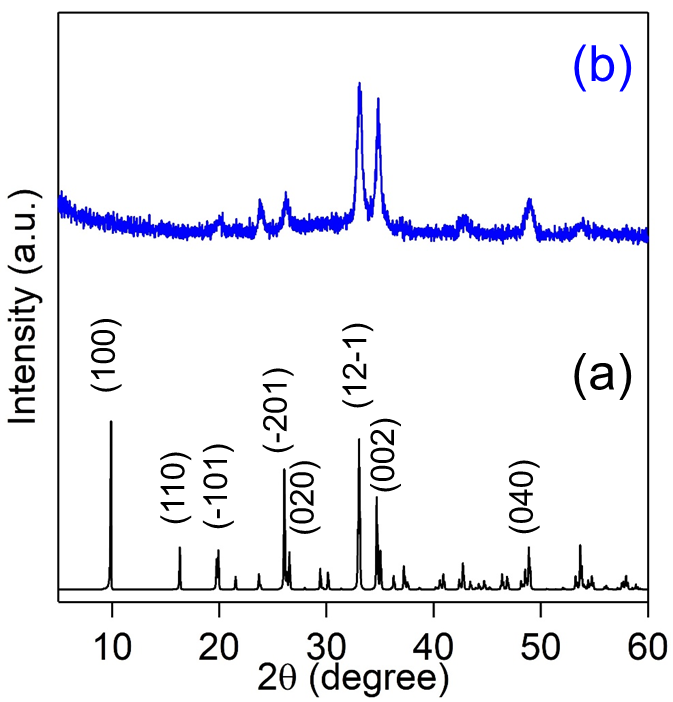


Figure S2. XRD patterns of Na_3_FePO_4_CO_3_ with refined parameters (a), and MM_NFPC (b).


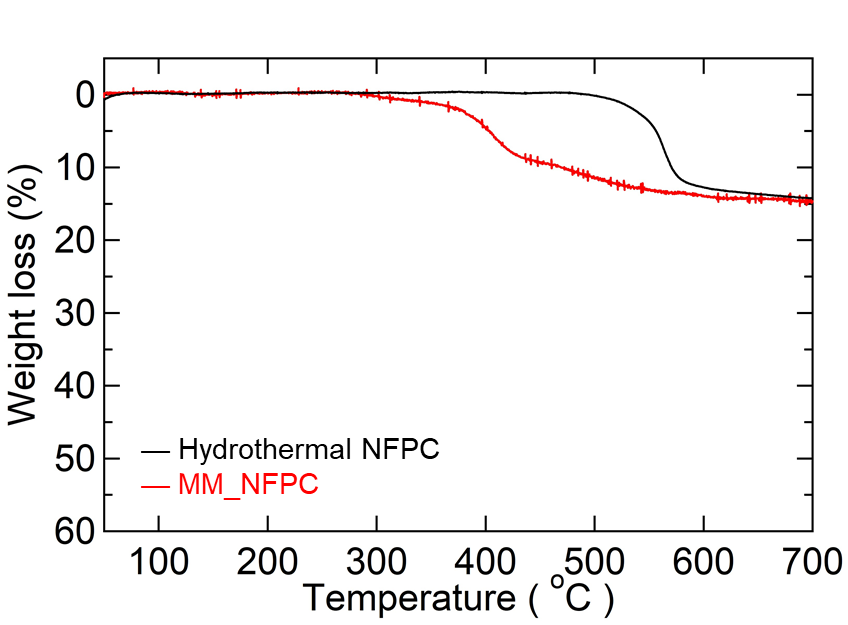


Figure S3. TG profiles of Na_3_FePO_4_CO_3_ synthesized by the mechanical ball milling method (red line) and hydrothermal method (black line).


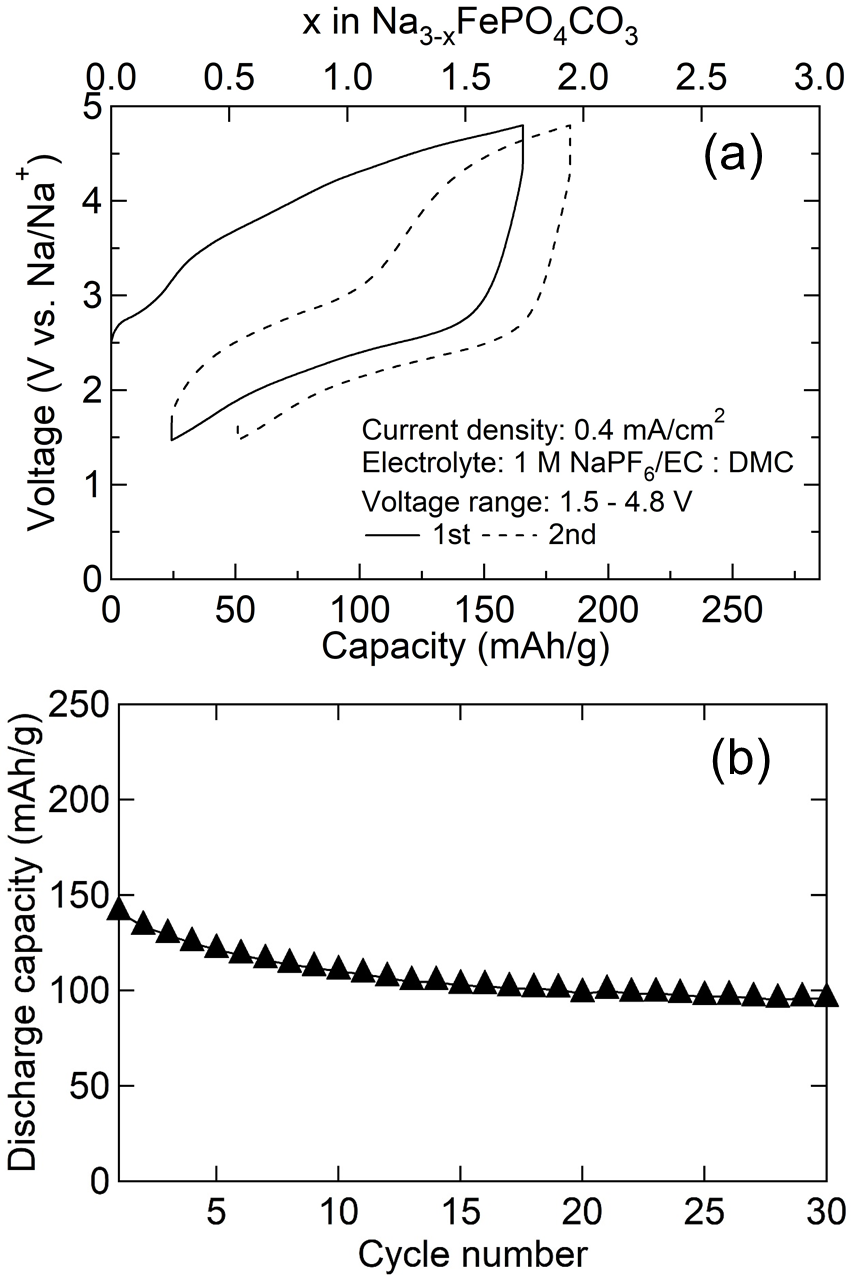


Figure S4. The initial charge-discharge curves obtained between 1.5 and 4.8 V at a current density of 0.4 mA/cm^2^ at room temperature (a); the cyclability is on the bottom side (b).


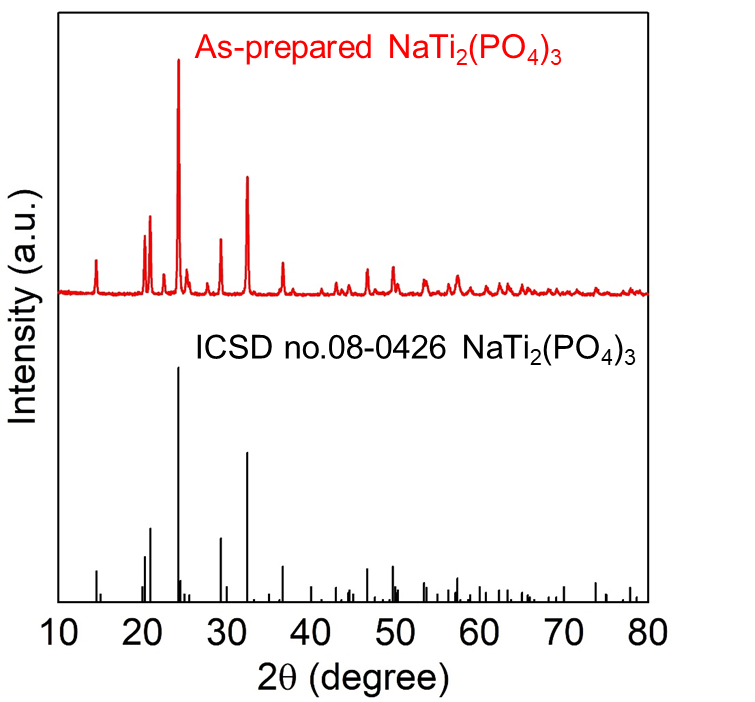


Figure S5. XRD patterns of NaTi_2_(PO_4_)_3_ prepared by solid state reaction.
